# A computational model to investigate the effect of pennation angle on surface electromyogram of *Tibialis Anterior*

**DOI:** 10.1371/journal.pone.0189036

**Published:** 2017-12-07

**Authors:** Diptasree Maitra Ghosh, Dinesh Kumar, Sridhar Poosapadi Arjunan, Ariba Siddiqi, Ramakrishnan Swaminathan

**Affiliations:** 1 Non-Invasive Imaging and Diagnostics Laboratory, Biomedical Engineering Group, Applied Mechanics Department, Indian Institute of Technology Madras, Chennai, India; 2 Biosignals Lab, School of Engineering, RMIT University, Melbourne, Australia; 3 Department of Electronics and Instrumentation, SRM University, Chennai, India; University of Houston, UNITED STATES

## Abstract

This study has described and experimentally validated the differential electrodes surface electromyography (sEMG) model for tibialis anterior muscles during isometric contraction. This model has investigated the effect of pennation angle on the simulated sEMG signal. The results show that there is no significant effect of pennation angle in the range 0° to 20° to the single fibre action potential shape recorded on the skin surface. However, the changes with respect to pennation angle are observed in sEMG amplitude, frequency and fractal dimension. It is also observed that at different levels of muscle contractions there is similarity in the relationships with Root Mean Square, Median Frequency, and Fractal Dimension of the recorded and simulated sEMG signals.

## Introduction

Tibialis Anterior (TA) is essential for posture and gait stability. Age-associated weakness of TA is known to be a major cause of falls [1–3]. As there is no direct way for measuring TA contraction force, sEMG provides a useful tool to measure the electrical activity of the muscle associated with the force of contraction.

Numerical modelling has been considered to improve the interpretation of the relationship between neuromuscular parameters, generated force and sEMG signal [4] and these models have demonstrated the relationship of many parameters with the signal [5–9]. However, their applications are very limited because of number of assumptions involved, such as the parallel orientation of muscle fibres to the surface [9]. These assumptions make the models unsuitable for Tibialis muscles which are pennate. In pennate muscles, fibres run at an angle to the axis of traction. The anatomical cross sectional area (ACSA) does not represent the cross section perpendicular to all fibres in the muscle, i.e., the physiological CSA (PCSA) [10]. The maximum force of a muscle depends on its PCSA rather than its ACSA [10, 11].

Modelling sEMG of TA requires consideration of pennation angle of the muscle [11]. SEMG signals are recorded using differential electrodes as it enhances the signal to noise ratio [12]. However, some researchers have investigated pennate muscles [13–17], they have not considered the differential electrodes and made assumptions on the shape of the signals [18]. While the pre-defined shape of MUAP is reasonable for muscles such as biceps, it may lead to erroneous results because of the possible age-associated change in muscle shape due to pennation angle in TA. It is reported that power spectrum depend on shape of MUAP [19, 20]. Howard et al [21] reported that properties of action potential changes due to aging. Hence, for the model to be effective for investigating changes to sEMG with ageing or disease, it is essential for the model to provide the examiner the accurate estimate of the shape of SFAP.Zuniga et al [22] reported that there is no significant difference in the EMG amplitude and mean power frequency MPF responses for EMG recorded from the electrode oriented parallel and perpendicular to the muscle fibres of the vastus lateralis muscle (VL). Shi et al [23] reported exponential relationship between the RMS and pennation angle of brachialis. These studies have considered only the amplitude and spectral based features and have not investigated the features describing its chaotic nature. However, muscle contractions are reported to exhibit fractal characteristics [24, 25]. We report a computational model that describes the generation of sEMG signals for TA muscle where we have investigated the effect of pennation angle on sEMG recorded using differential electrode. The model has been validated experimentally based on the temporal, spectral and chaotic signal properties by comparing simulated and experimental recordings using the three features; Root Mean Square (RMS), Median Frequency (MDF) and Fractal Dimension (FD).

## Materials and methods

### Description of the model

The model is based on the works reported by Wheeler et al [9] and Siddqi et al [18] with two significant enhancements; i) differential electrodes and ii) pennation angle. The use of differential electrodes overcomes the need for pre-defined shape of Motor Unit Action Potentials (MUAP), while the second allows the investigation of muscles such as TA.

#### Volume conduction

**Fig 1:**
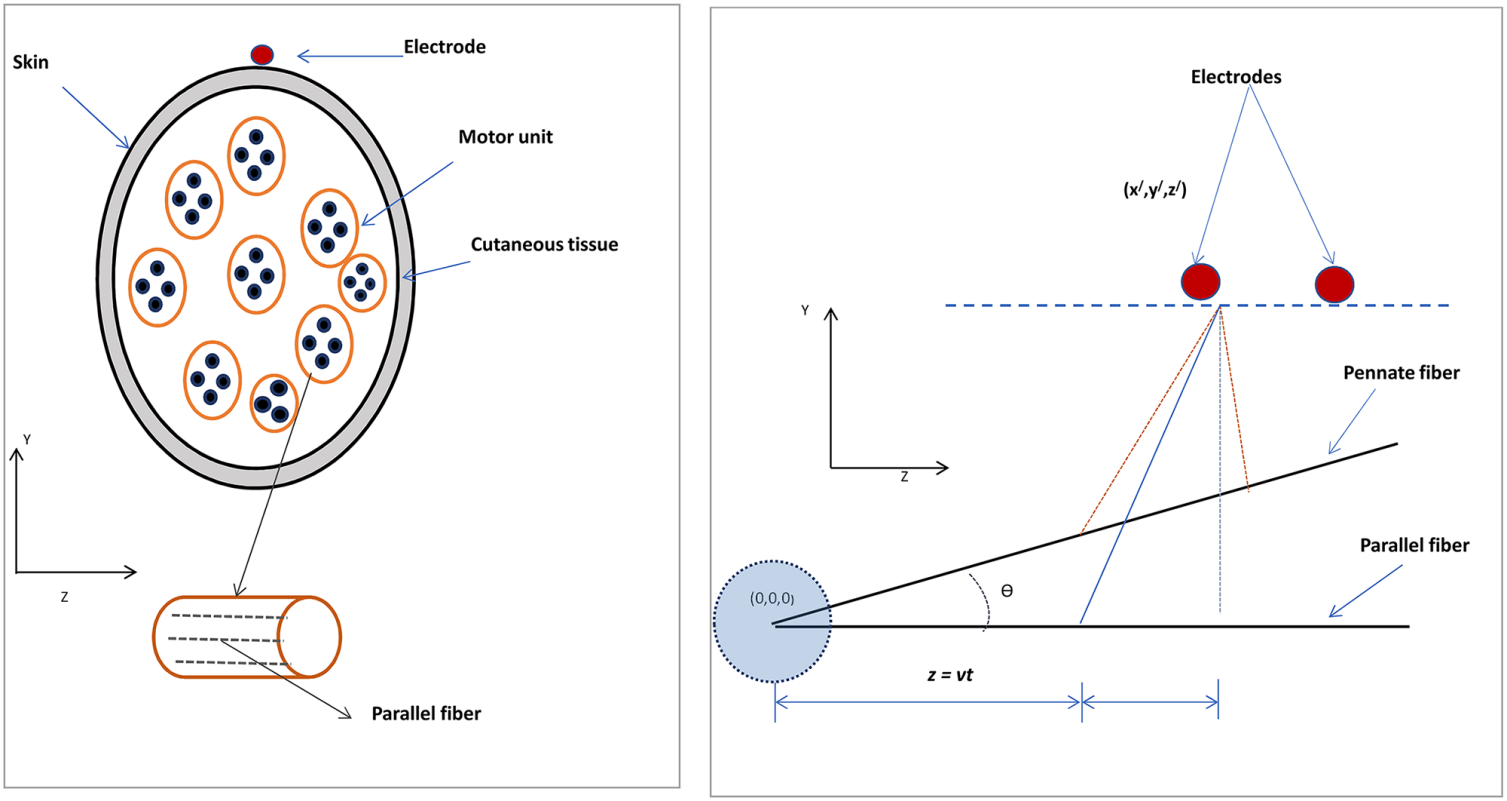
Representation of EMG model: a) position of motor units of muscle with respect to electrode located at skin, b) position of parallel fibre and pennate fibre with respect to electrode.

The motor-unit territory cross-section was considered to be circular and described using Cartesian coordinate system located at the centre of muscle. The cross-section area was obtained based on the type of muscle fibres and number of fibres in the muscle. The electrodes were separated from the muscle by two layers; skin and subcutaneous [7]. The innervation zone was located at the centre of the fibre length. The model is described in Fig 1.

Muscle fibre action potential was calculated by Rosenfalk Eq 1 [8, 26]. The current distribution between the inside and outside of the muscle cell was modelled as the second spatial derivative of action potential voltage V_m_ [27] given in Eq 2.

$${\text{V}}_{\text{m}}\left(\text{Z}\right)=\text{A}{\left(\lambda \text{Z}\right)}^{3}{\text{e}}^{-\lambda \text{Z}}-\text{B}$$ (1)

$${\text{I}}_{\text{m}\;}\left(\text{t}\right)={\text{CA}\lambda }^{2}\left(\lambda \;\left(\text{vt}\right)\right)\left(6-6\lambda \;\left(\text{vt}\right)+\lambda^{\;2}{\left(\text{vt}\right)}^{2}\right){\text{e}}^{-\lambda \left(\text{vt}\right)}$$ (2)

where, I_m_(t) is trans membrane current, C is a constant and is given as $\frac{d^{2}\sigma_{i\;}\pi }{4v^{2}}$, $\sigma ={\left(\frac{\sigma_{i}}{\sigma_{e}}\right)}^{2}$, σ_i_ is internal conductivity, σ_e_ is external conductivity, d is fiber diameter, v is the conduction velocity, A is the amplitude of action potential, B is resting membrane potential, λ is a scaling factor, z_e_ is the distance between the electrode and neuromuscular junction and σ is assumed to be direction independent.

Earlier models [9, 18] have considered a single electrode with a pre-defined tri-phasic motor unit action potential. However, such models are limited because these cannot be used to investigate the effect of electrode placement, inter-electrode distance and pennation angle. To overcome these limitations, the model described in this paper has considered differential electrodes and the TA muscle has been modelled as uni-pennate structure [18] with angle θ to the surface. Thus, Eq 3 which describes the volume conduction effect in the muscle has been modified to Eq 4.

$$\text{f}{\left(\text{t}\right)}_{\text{Parallel}}=\frac{1}{4\pi \sigma_{\text{e}}\sqrt{{\left(\text{z}-\text{z}\prime \right)}^{2}+[\sigma {\left(\text{x}-\text{x}\prime \right)}^{2}+{\left(\text{y}-\text{y}\prime \right)}^{2}]}}$$ (3)

$$\text{f}{\left(\text{t}\right)}_{\text{Pennate}}=\frac{1}{4\pi \sigma_{\text{e}}\sqrt{{\left(\text{z}-\text{z}\prime \text{cos}\theta -\text{y}\prime \text{sin}\theta \right)}^{2}+[\sigma {\left(\text{x}-\text{x}\prime \right)}^{2}+{\left(\text{y}\prime \text{cos}\theta -\text{z}\prime \text{sin}\theta \right)}^{2}]}}$$ (4)

In these equations, the neuro-muscular junction is considered as point of origin (0, 0, 0), x, y, z is the location of the action potential and x’, y’, z’ is the location of the electrode ‘0’ and x’_1_, y’_1_, z’_1_ of electrode ‘1’. The muscle fibres are aligned along z direction.

#### Motor unit recruitment and rate coding

In the model described in this paper, the motor-units (MU) are recruited according to the size principle [28]. All the motor units are recruited at a given force recruitment range (RR) according to Fuglevand model [29]. RR is expressed in percentage of MVC.

**Fig 2:**
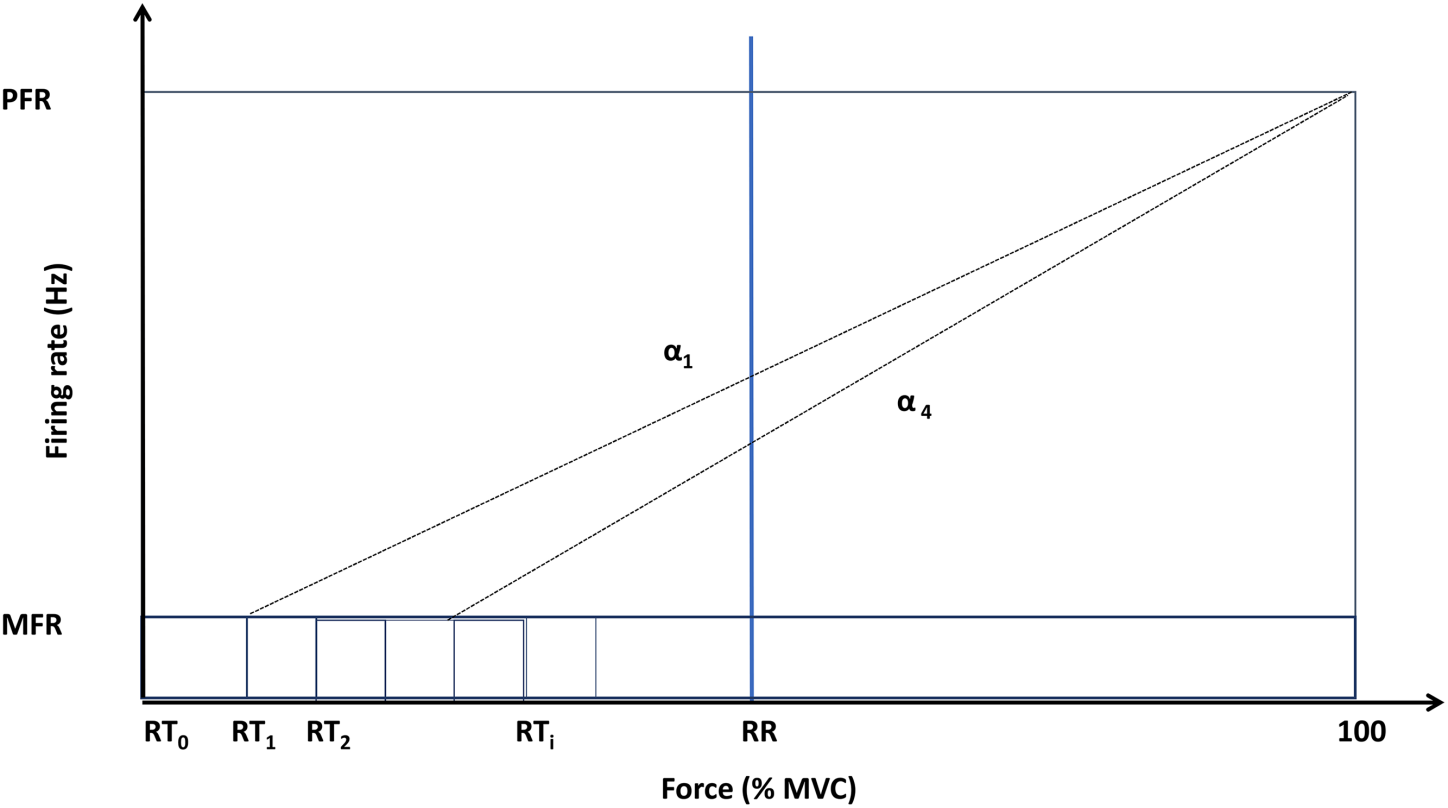
Schematic diagram representing the relation between firing rate and muscle force (MVC).

The i^th^ MU is activated once the neural drive crosses its recruitment threshold (RT_i_) which is based on the force recruitment range (RR) based on Fuglevand model [29].

$${\text{RT}}_{\text{i}}=\text{exp}\left(\frac{\text{ln}\text{RR}}{\text{TMu}}\right)\text{i}$$ (5)

where TMu is the total number of motor units and ‘i’ is the motor unit index based on its size rank. RR is the recruitment range and is assumed to be the voluntary input based on fraction of MVC. The firing rate is controlled by input force RR shown in Fig 2 and given in Eq 6 (3).

Firing rate of active motor-units has been considered to increase linearly from Minimum Firing Rate (MFR) to Peak Firing Rate (PFR) as a function of the fraction of maximum voluntary contraction of the muscle. It is assumed that all motor units reach their PFR at 100% MVC. MFR ranges from 7 to 23 Hz while PFR ranges from 14 to 50 Hz for human skeletal muscles (5). This has been summed in Eqs 6 and 7;

$$FR_{i,RR}=(RT_{RR}-RT_{i})\alpha_{i}+MFR$$ (6)

$$\alpha_{i}=\frac{\left(PFR-MFR\right)}{\left(100-RT_{i}\right)}$$ (7)

where FR_i,RR_ represents the firing of i^th^ motor unit at input force level RR and α_i_ represents the slope associated with i^th^ motor unit;

### Model simulation

The values of the parameter were taken from experimental studies reported in the literature and are listed in Table 1 [30]. The model was simulated 20 times with values randomly obtained from the range shown in Table 1 such that it represented more realistic values and was comparable with the experimental data.

**Table 1:** Model parameters applied in the simulation study.

Parameter	Value
Number of motor units [31]	125–652
Muscle fiber diameter Type 1(μm)[32] (43)	35.46
Muscle fiber diameter Type 2 ((μm) [32]	50.68
Muscle radius (mm) [33]	20±2
Slow fiber Conduction velocity (m/s)	3.9± 0.3
Fast fiber Conduction velocity (m/s) [34]	4.9± 0.3
Muscle half fiber length (mm) [35]	45±0.4
Mean firing rate (Hz) [5]	7–23
Peak firing rate (Hz) [5]	14–50
Pennation angle (degree) [35]	20±2
Cutaneous tissue (mm) [36]	3

### Experiments

Five young volunteers with age—range: 23–30 years Mean (±SD): 26.1 ±2.9 years and body mass index—range: Mean (±SD): 22.3 Kg/ m^2^ +2.9 with no clinical or self-reported history of neuromuscular disease or ankle injury participated in this study. Seven participants were approached, and two subjects did not participate in the study. All research involving human participants has been approved by Royal Melbourne Institute of Technology (RMIT) University College Human Ethics Advisory Network (CHEAN) committee and all clinical investigations have been conducted according to the principles expressed in the Declaration of Helsinki. Prior to the participation in the experiment, written informed consent was obtained from the participants.

The experimental set-up and protocol for recording surface Electromyography used in this study have been reported in Siddiqi et al [18]. SEMG activity was recorded from TA (Fig 1) muscle using SENIAM configuration. The ground electrode was placed at the patella. The locations for electrodes were shaved, abraded and cleansed with an alcohol swipe to ensure good connectivity. The signals were recorded using the Delsys Myomonitor 4 (Delsys, Boston) with fixed gain of 1000, CMRR of 92 dB and bandwidth of 2–450 Hz, with 12 dB/ octave roll-off. The sampling frequency was 1000 Hz with a resolution of 16 bits/ sample. The Delsys single-channel active differential surface electrodes with an embedded preamplifier and inter-electrode distance of 10 mm were used.

Participants sat with their right leg strapped, hip at 90°, knee at 140° and ankle at 90°. SM100-type strain gauge force sensor (Interface S type) was used to immobilize the foot-plate and measure the isometric force produced during dorsiflexion or plantar flexion. Its output was displayed to give visual feedback to the subject for maintaining steady contraction. Participants performed an isometric contraction at 25%, 50%, 75% of their maximum voluntary contraction (MVC) for 15 seconds each. Throughout the experiment, the left leg was planted firmly on the ground. Absence of heel lift, and foot or toe movement during plantar flexion and dorsiflexion was ensured with the foot and ankle secured to the footplate.

### Data analysis

The experimentally recorded and simulated sEMG signals were analysed to obtain three features; RMS, MDF and FD. While most of the earlier studies have investigated the RMS and MDF of simulated sEMG, these suffer from large inter-subject variation which makes it difficult for validating the model based on experimental recordings. FD is an important feature and is an intrinsic property of a muscle [37, 38] and has lower variability compared with other parameters. To determine the relationship of the angle of pennation with the signal property, the model was simulated for different values of θ and single fibre action potential (SFAP) was obtained. Correlation analysis was conducted to identify the similarity between SFAP for fibres with θ pennation angle with the parallel fibres (θ = 0). Consider sEMG with parallel muscle fibres is *x* and sEMG corresponding to fibres with pennation angle of θ is *y*; then *r*_*xy*_ = Sample correlation coefficient, *s*_*xy*_ = Sample covariance, *s*_*x*_ = Sample standard deviation of *x*, *s*_*y*_ = Sample standard deviation of *y* Correlation coefficient, given by

$${\text{r}}_{\text{xy}}=\frac{{\text{S}}_{\text{xy}}}{{\text{S}}_{\text{x}}{\text{S}}_{\text{y}}}$$ (8)

The following features of sEMG were computed to compare the simulation and experimental data:

1. Root Mean Square (RMS),
  $$\text{RMS}=\sqrt{\frac{\sum_{i=1}^{N}x_{i}^{2}}{N}}.$$ (9)
  *x*_*i*_ represents the *i*^th^ sample of the signal while *N* is the window size which was taken to be 2500 ms based on [39][33].
2. Median Distribution Frequency (MDF):
  $$\sum_{j=1}^{MDF}P_{j}=\sum_{j=MDF}^{M}P_{j}=\frac{1}{2}\sum_{j=1}^{M}P_{j}$$ (10)
  *P*_*j*_ is the power spectrum at frequency bin *j* and *M* is length of the frequency bin(33).
3. Higuchi’s Fractal Dimension (FD): FD was computed using the algorithm described in [40, 41] and briefly described below.

Considering *y* (*n*) is the signal for epoch *n*, *n = 1 to* N, and this is sub-divided in *k* segments,$y_{m}^{k}$

$$y_{m}^{k}=\{y(m),y(m+k),y(m+2k),\ldots .y(m+\left\lfloor \frac{N-m}{k}\right\rfloor k)\}\;\text{m}=0,1,2\ldots .,\text{k}$$ (11)

where *m* represents the initial time and *k* is interval time.

$\left\lfloor \frac{N-m}{k}\right\rfloor$ denotes integer part of $\frac{N-m}{k}$

The average length L_m_(k) is calculated as

$$L_{m}(k)=\frac{\sum_{i=1}^{\left\lfloor (N-m)/k\right\rfloor }\left|y(m+ik)-y(m+(i-1)k\right|(n-1)}{\begin{array}{cc} \frac{\left\lfloor N-m\right\rfloor }{k} & k \end{array}}$$ (12)

The total length of the entire epoch in *k* time interval is calculated as

$$\text{L}\left(\text{k}\right)=\sum_{\text{m}=1}^{\text{k}}{\text{L}}_{\text{m}}\left(\text{k}\right)$$ (13)

*L(k)* is proportional to *k*^*-D*^ for total average length for scale k. D is the FD in Higuchi’s method. The slope of the least squares linear best fit of the curve *ln(L(k))* versus *ln(1/k)* estimates the fractal dimension.

Tuning of k parameter is necessary in Higuchi’s algorithm because the correct selection of k parameter has an important role to obtain reliable FD values [41](36). In this work, k = 6 is used to get the best estimate of fractal dimension [40, 41]. Correlation analysis was performed to identify the similarity in the shape of the SFAP for different pennation angles.

#### Statistical testing

a) Paired sample t-test: RMS, MDF and FD are expressed as mean (standard deviation), and compared using two-sample t-test for sEMG signals simulated and experimental data. The differences were considered significant at *p*<0.05.
b) Two One Sided Test (TOST): The similarity of the experimental and simulation data was tested using Two One Sided Test (TOST) [42]. The test was performed for the three levels of MVC and for the three features independently; RMS, MDF and FD. Bland Altman plots are used to select the lower bound and upper bound for TOST.

## Result

**Fig 3:**
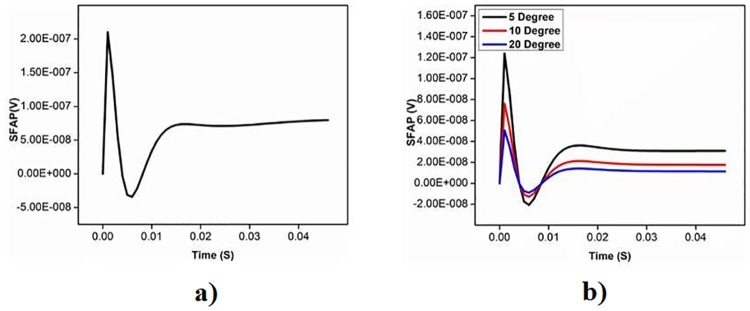
The SFAP with differential electrode a) parallel fibre b) pennate fibre.

**Fig 4:**
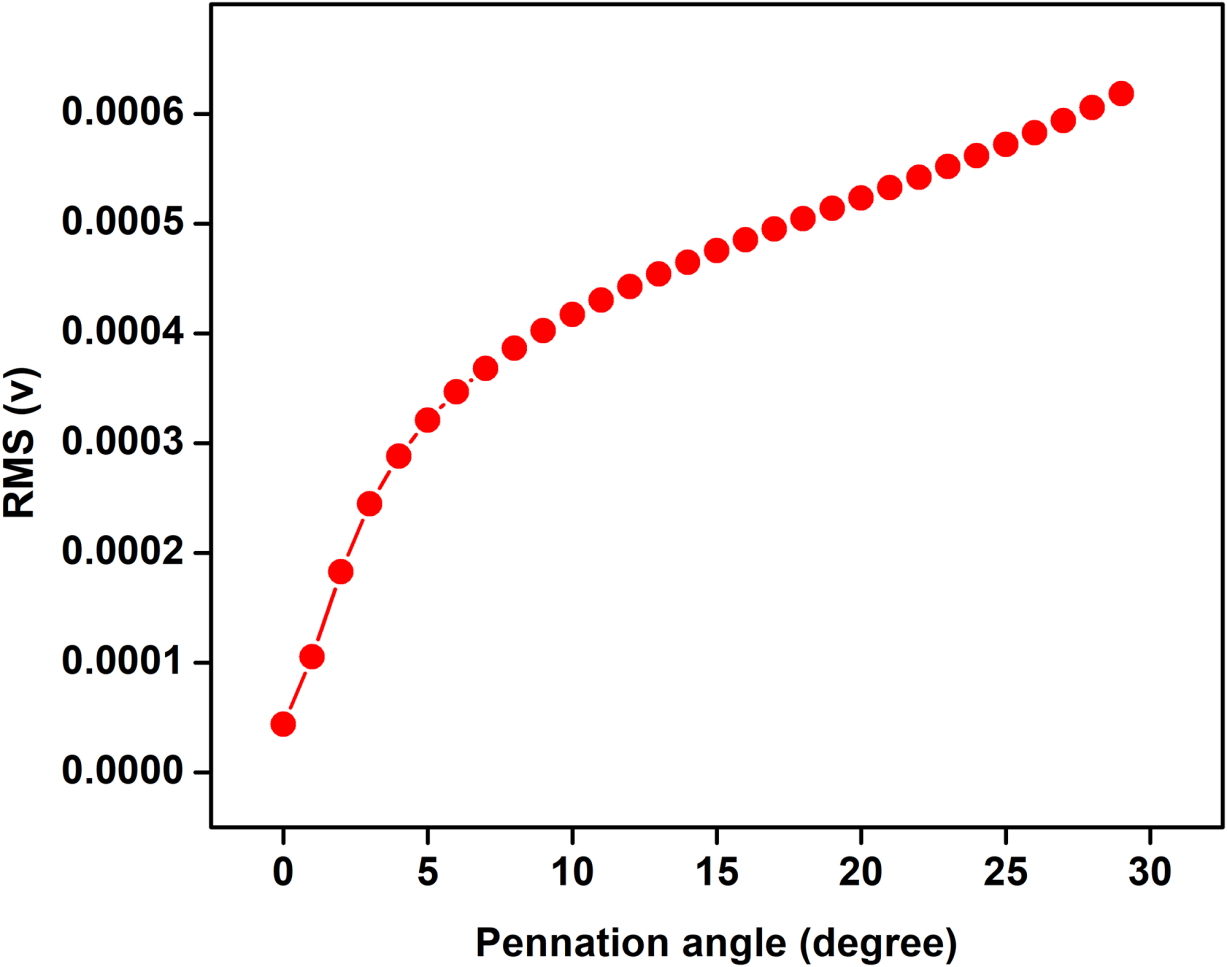
Change in sEMG amplitude with variation in pennation angle.

**Fig 5:**
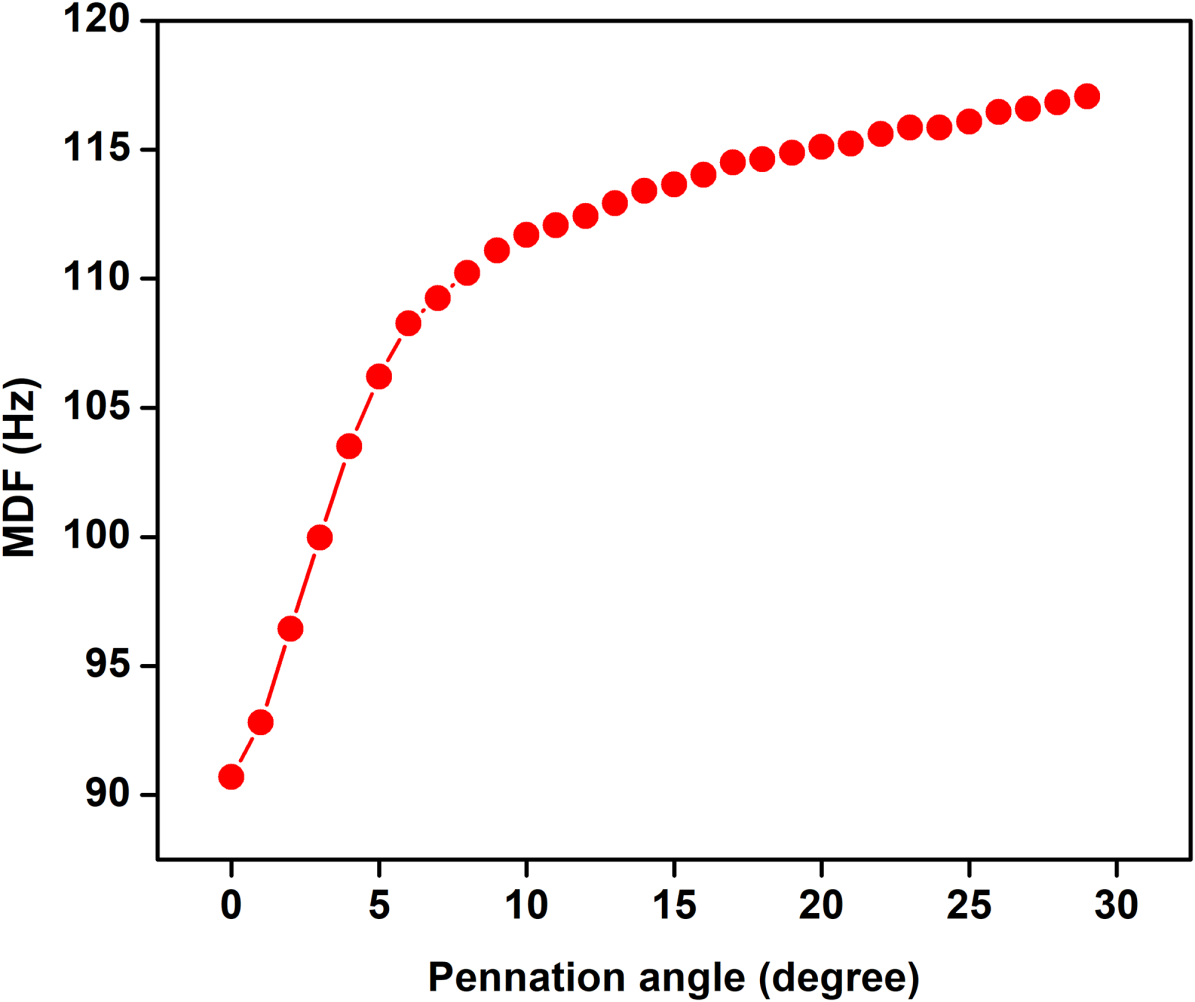
Change in sEMG frequency with variation in pennation angle.

**Fig 6:**
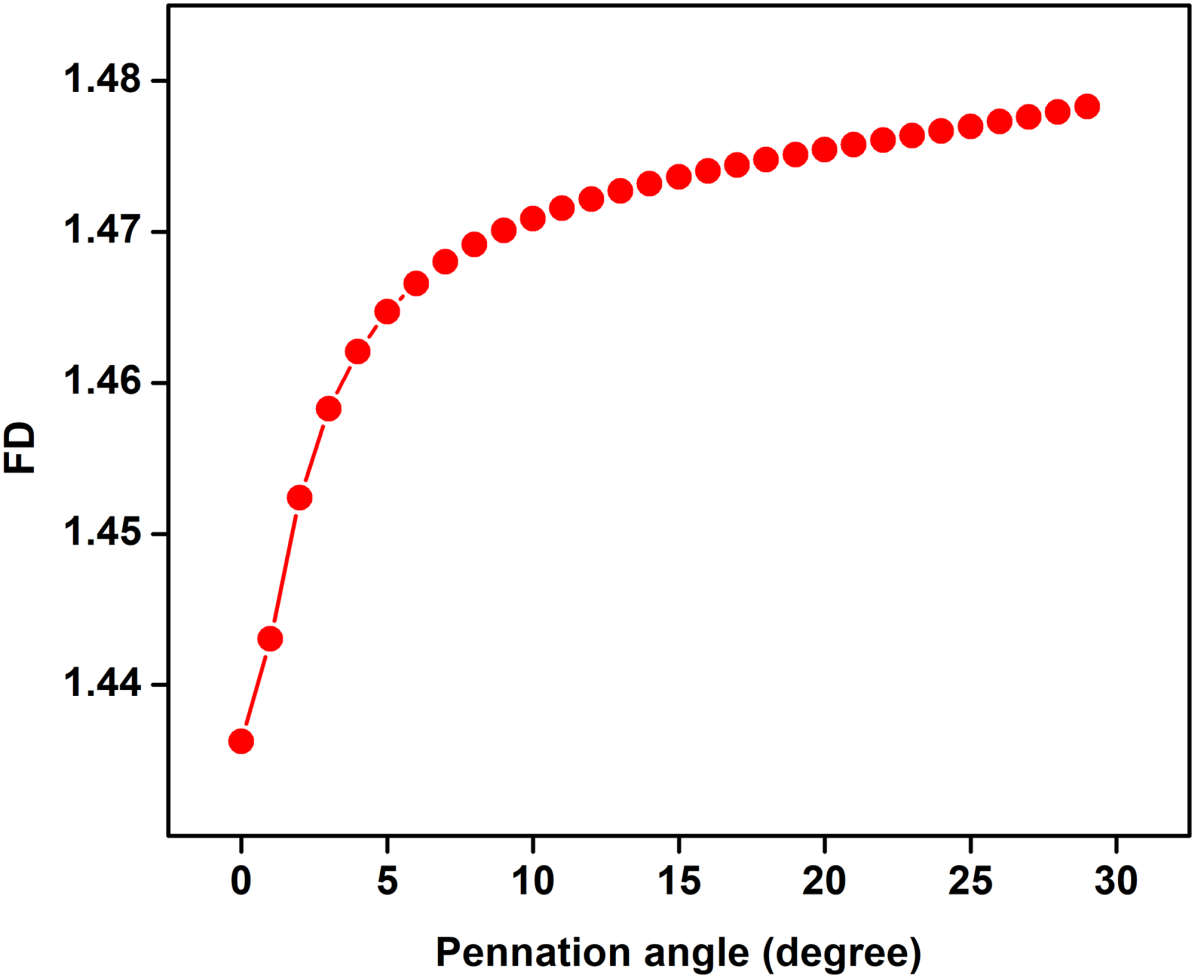
Change in sEMG fractal dimension with variation in pennation angle.

The effect of pennation angle on the shape of simulated single fibre action potential (SFAP) for θ ranging from 0 to 20 degrees is shown in Fig 3. The pennation angle is increased up to 20 degree because maximum pennation angle for TA is reported 20 degree in literature [22] (22). It is observed that the shape of SFAP is similar and triphasic for all the four values of θ. Table 2 represents the correlation coefficients and P values between shape of SFAP of parallel muscle and pennate muscle with different pennation angle. It is observed that the correlation is high for all the angles in the range (r>0.9). The relationship of pennation angle with amplitude, frequency and fractal dimension (FD) of sEMG is shown in Figs 4–6 respectively. From these figures, it is observed that amplitude, frequency and FD of sEMG increase with increased pennation angle, while the shape remains unchanged.

**Table 2:** Correlation coefficient comparing the shape of single fibre action potential between parallel and pennate fibre of different angles.

Pennate Fiber	Correlation coefficient	P-value
0 degree (parallel fiber)	1.0	1.0
5 degrees	0.9909	0.6785
10 degrees	0.9711	0.699
20 degrees	0.9208	0.493

**Fig 7:**
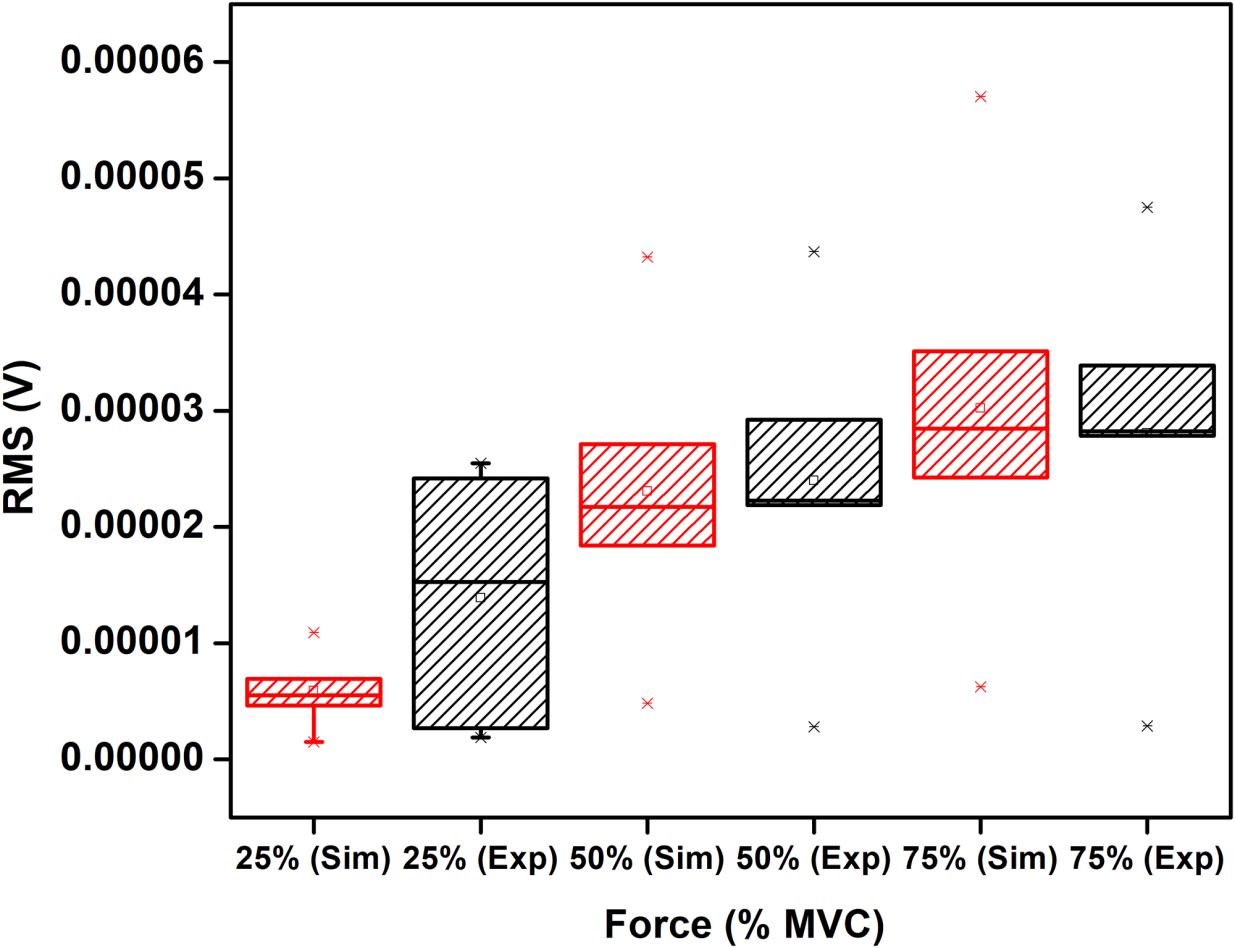
Comparison between RMS of simulated and experimentally obtained sEMG for 25%, 50% and 75% MVC. The circle and asterisk in each data set indicate mean and outlier respectively.

**Fig 8:**
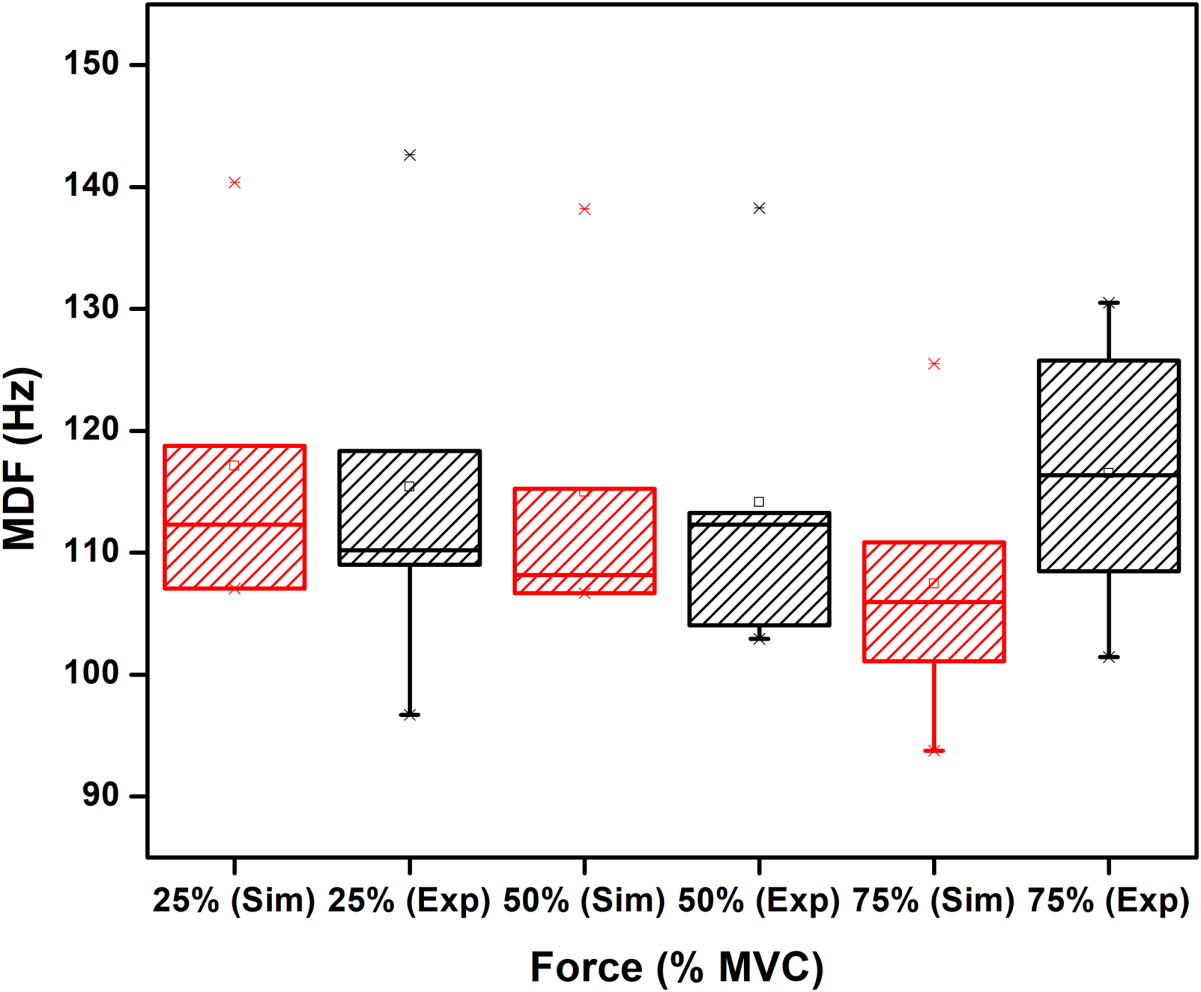
Comparison between MDF of simulated and experimentally obtained sEMG for 25%, 50% and 75% MVC. The circle and asterisk in each data set indicate mean and outlier respectively.

**Fig 9:**
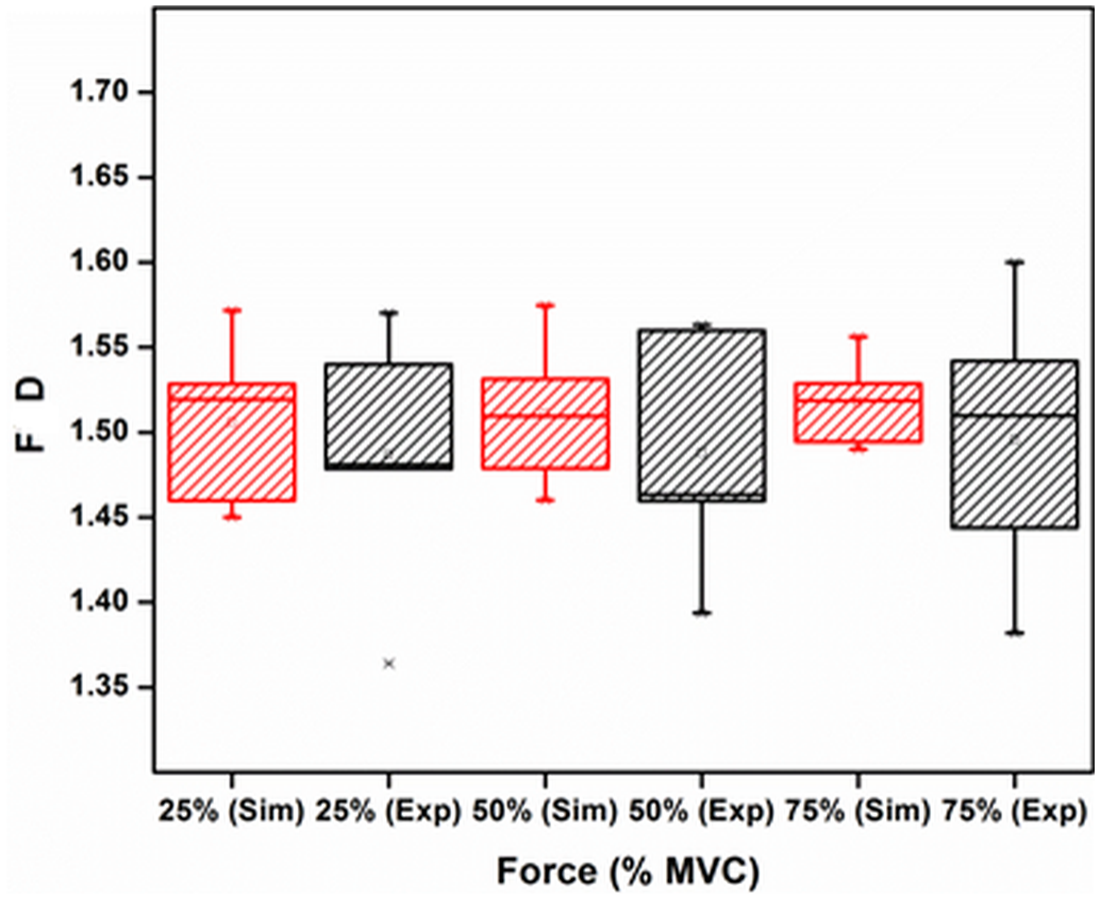
Comparison between FD of simulated and experimentally obtained sEMG for 25%, 50% and 75% MVC. The circle and asterisk in each data set indicate mean and outlier respectively.

Table 3 shows the mean values of RMS, MDF and FD for the three different levels of %MVC for simulated and experimental data. Two sample t-test is performed between experimental and simulated data for each level of MVCS and corresponding p-values are given. From the Box-whisker plots in Figs 7–9, it is observed that while the three features of the experimental and simulated data follow similar trends, one common difference is that the experimental data has larger range of the quartiles compared with the simulated data.

**Table 3:** Comparison of mean and SD between simulated experimental sEMG signals and statistical t-test for Tibialis Anterior muscle.

F.No.	Feature	%MVC	Simulated		Experimental		p-value
			Mean	SD	Mean	SD	
1	RMS	25%	5.88e-06	3.43e-06	1.38e-05	1.13e-05	0.169
		50%	2.30e-05	1.3e-05	2.39e-05	1.47e-05	0.922
		75%	3.02e-05	1.84e-05	2.80e-05	1.61e-05	0.850
2	MDF	25%	117.11	13.87	115.37	17.07	0.86
		50%	114.98	13.44	114.14	14.26	0.925
		75%	107.42	11.91	116.49	11.978	0.26
3	FD	25%	1.50	0.05	1.48	0.078	0.66
		50%	1.51	0.04	1.48	0.072	0.566
		75%	1.51	0.02	1.49	0.08	0.59

**Fig 10:**
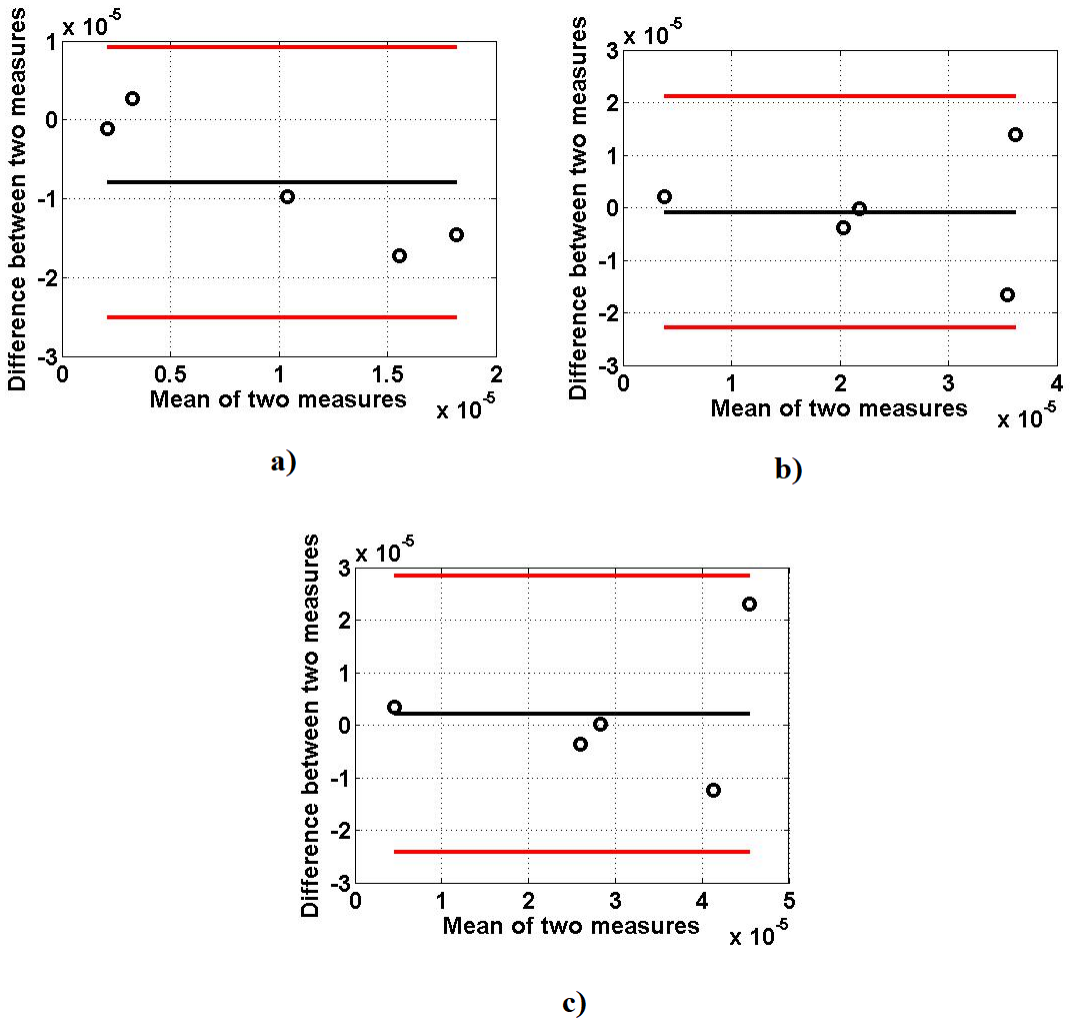
Bland Altman plots between RMS of simulated and experimentally obtained sEMG signals: (a) 25% MVC, (b) 50% MVC (c) 75% MVC.

**Fig 11:**
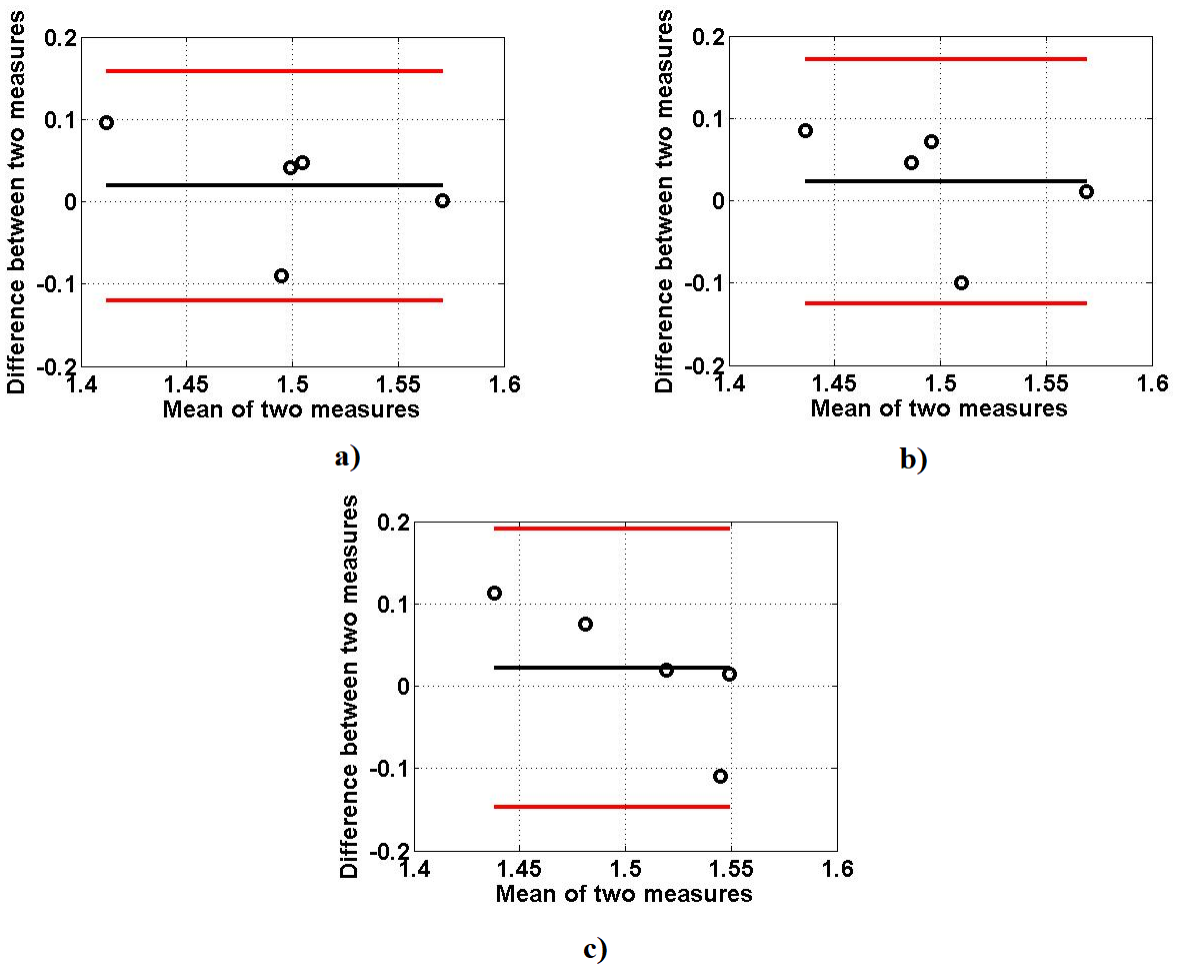
Bland Altman plots between FD of simulated and experimentally obtained sEMG signals: (a) 25% MVC, (b) 50% MVC (c) 75% MVC.

**Fig 12:**
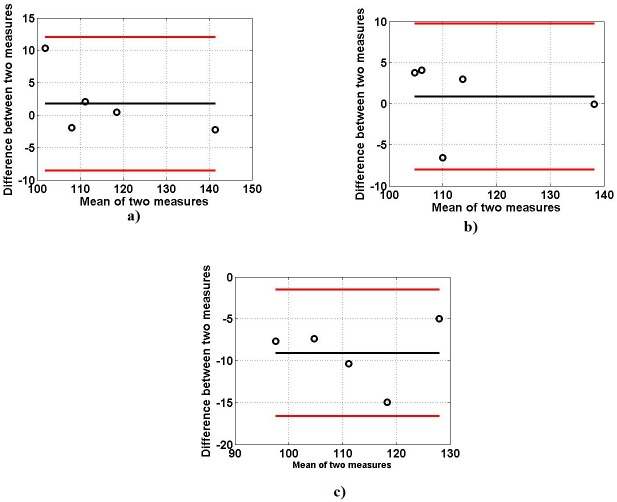
Bland Altman plots between MDF of simulated and experimentally obtained sEMG signals: (a) 25% MVC (b) 50% MVC (c) 75% MVC.

The results of the similarity test performed by TOST are shown in Table 4. To select the lower and upper boundaries for equivalent test, Bland Altman plots are given for each feature in different MVCs as shown in Figs 10–12. This test shows that there is similarity between the two data sets; simulated and experimental, within the intervals (±2 SD) at 90% confidence interval as observed in Bland Altman plots. The corresponding ‘p’ values shown in the Table 4 indicate that the two data are significantly similar within the range.

**Table 4:** Statistical equivalence test for simulated and experimental values for RMS, MDF and FD.

RMS		FD		MDF	
MVC	P-value	Test	Value	Test	Value
**25%**	0.006	**25%**	0.005	**25%**	0.046
**50%**	0.020	**50%**	0.0026	**50%**	0.045
**75%**	0.023	**75%**	0.001	**75%**	0.049
**Test interpretation**	Equivalent	**Test interpretation**	Equivalent	**Test interpretation**	Equivalent

## Discussion

We have investigated the effect of pennation angle on the shape of SFAP and observed that there is no significant difference between the signals for θ ranging from 0 to 20°. This supports the approximation which was proposed by Siddiqi et al [18]where TA had been approximated to be parallel fibre muscles. Amplitude of sEMG increases as pennation angle increases from 0 degree to 30 degree and this is in line with literature [23]. Studying the effect of pennation angle is important when comparing sEMG recorded from young and older cohort since it is known that pennation angle changes with age for pennate muscle [11]. However, this analysis suggests that there is no change in the shape of the SFAP with pennation angle, and this would indicate that the age-associated change in sEMG is not due to this factor.

From Figs 7–9 and Table 3, it is observed that the trend of RMS, MDF, FD of simulated and experimentally recorded sEMG are similar. While the RMS results are in agreement with previous studies [43, 44] for biceps brachii muscle and as has been discussed by Moritani et al [45], no earlier study has examined the FD of simulated sEMG.

The results show that while the trends of MDF of the simulated and experimental recordings with respect to the fractional MVC are similar, the absolute values are different. There may be number of reasons that could contribute to this difference. For example, the spectrum of sEMG is highly dependent on the electrode placement and inter-electrode distance [46] and hence comparing the absolute values of the MDF may be difficult.

One common difference between the simulated and experimental data is that the quartile range for experimental is much higher than the simulated. Moreover, this study has considered the parameters to belong to a range of values rather than single value and repeated simulations have been initialized randomly within the range, the results indicate that the actual variation appears to be larger. This may be because we have assumed normal distribution which would be require very large number of experiments while this study reports the data from five participants.

This study has investigated the FD of simulated and experimentally recorded sEMG. While the trends of the two are similar, there is a difference in the absolute values. The FD corresponding to the experimental data is higher than that of the simulated data. It is known that the Higuchi’s FD is dependent on the complexity of the signal sources and this difference may be attributable to the necessity of models being simplified versions of the real system.

The study has some limitations as it has experimentally tested only with small number of healthy participants. The model reported in this study is two- dimensional, and future studies will investigate the influence of physiological conditions such as aging and disorder on the skeletal muscle system with large number of participants.

## Conclusions

This study has developed a computational model that describes sEMG suitable for pennate muscles. The model was validated for the TA muscle by comparing the experimental with the simulated sEMG for different fractions of MVC. Three features were considered; RMS, MDF and FD. The results show that while the relationship between these features and fraction of MVC are similar between simulated and experimental data, there is difference in the absolute values. This indicates that the simulated values are subjected to normalization and while it can be used to predict the trends, but is unsuitable for predicting the absolute values of the recording.

The model has investigated sEMG for different muscle fibre types, pennation angle and the results show that there is no significant difference in the shape of SFAP because of change in the pennation angle in the range of 0 to 20°. However, the changes were observed in amplitude, frequency, and chaotic nature of sEMG signals. This reveals that parallel model of the TA can be used to approximate the TA, and this eliminates the need of computationally intensive volume conductor model with angle of muscle fibres to represent the muscle.
